# Lipidomics-based investigation of its impact on the pathogenesis of coronary atherosclerosis: a Mendelian randomization study

**DOI:** 10.1186/s41065-025-00367-x

**Published:** 2025-02-01

**Authors:** Qun Wang, Yuan Cao, Lianqun Jia

**Affiliations:** Key Laboratory of Ministry of Education for TCM Viscera-State Theory and Applications, Ministry of Education of China, Liaoning University of Traditional Chinese Medicine, 79 Chongshan Road, Huanggu District, Shenyang, 110847 Liaoning Province China

**Keywords:** Mendelian randomization, Lipid metabolism, Atherosclerosis, Intermediate analysis, Incidence of a disease

## Abstract

**Background:**

Considerable attention has been devoted to investigating the association between lipid metabolites and cardiovascular diseases, particularly coronary atherosclerosis.

**Methods:**

A two-sample MR framework was used to investigate the relationship between lipid metabolites and the risk of coronary atherosclerosis. Two GWAS datasets were examined to take intersections of SNPs from 51,589 cases and 343,079 controls, and 14,334 cases and 346,860 controls to determine genetic susceptibility to coronary atherosclerosis. Random-effects inverse variance weighted (IVW) MR analyses were performed by a series of sensitivity assessments to measure the robustness of our findings and to detect any violations of MR assumptions.

**Results:**

Through IVW, MR-Egger and weighted median regression methods, we inferred that these six lipid metabolites: cholesterol levels, sterol ester (27:1/18:2) levels, triacylglycerol (52:4) levels, triacylglycerol (52:5) levels, diacylglycerol (18:1_18.2) levels, triacylglycerol (53:4), could directly impact the development of atherosclerosis.

**Conclusion:**

In conclusion, our study comprehensively illustrates a causal relationship between lipid metabolites and the risk of coronary atherosclerosis. Furthermore, cholesterol levels, sterol ester (27:1/18:2) levels, triacylglycerol (52:4) levels, triacylglycerol (52:5) levels, diacylglycerol (18:1_18.2) levels, and triacylglycerol (53:4) levels are positively correlated with the risk of coronary atherosclerosis. These six lipid metabolites have the potential as new predictors of the risk of atherosclerosis, providing new insights into the treatment and prevention of cardiovascular diseases.

## Background

Cardiovascular disease (CVD) ranks as the foremost factor behind global mortality, with an annual toll of around 18 million deaths (31% of all deaths). Atherosclerotic coronary heart disease is the primary contributor to CVD deaths, responsible for nearly 45% of all cases [1]. Coronary atherosclerosis is a long-term refractory disease with a wide range of clinical manifestations, from an asymptomatic state to stable angina, acute coronary syndrome (ACS), heart failure (HF), and sudden cardiac death (SCD) [2]. The principal issues in atherosclerosis are the local deposition of fat within arteries and the development of smooth muscle cells and fibrous matrix, which promote the formation of atherosclerotic plaques over time [3]. Studies have shown that atherosclerosis is characterized by inflammatory responses and arterial lipid accumulation [4]. Inflammation is an important driver of atherosclerosis [5] and atherosclerosis is a chronic inflammatory response that increases the risk of cardiovascular disease [6]. Lipid metabolites, including cholesterol and triglycerides, play significant roles in the development of inflammation. Cholesterol accumulation may promote inflammatory responses and exacerbate diseases associated with chronic metabolic inflammation, such as atherosclerosis and obesity [7]. Therefore, we speculate that inflammatory responses and lipid accumulation directly affect the risk of atherosclerosis.

Recent studies have shown that lipid abnormalities such as cholesterol and triglycerides are implicated in the pathogenesis of atherosclerosis [8]. Lipid metabolites include many types, like cholesterol, triglycerides, and phosphatidylcholine. Phosphatidylcholine is the most abundant phospholipid in all types of mammalian cells and subcellular organelles [9]. One study examined the plasma levels of TMAO biomarkers in Ldlr-/- male mice after dietary phosphatidylcholine supplementation and concluded that dietary phosphatidylcholine supplementation could improve atherosclerosis in mice [10]. Cholesterol is the major sterol in mammals and significantly affects membrane fluidity, permeability, and signaling [11]. All cell membranes require cholesterol, so cholesterol metabolism and its circulating levels are crucial for atherosclerosis [12]. Recent epidemiological data suggest that triglycerides are a causal pathway in the pathogenesis of atherosclerosis.

The important role of lipid metabolism disorders in the pathogenesis of atherosclerosis has been unveiled [13]. However, which lipid metabolites matter most has not been concluded. Therefore, in this study, MR analyses were performed with lipid metabolites as the exposure factor and coronary atherosclerosis as the outcome to explore the potential causal relationship. We aim to provide a theoretical basis for further research on the complex mechanisms and clinical efficacy of lipid metabolites in the risk of atherosclerosis.

## Methods

### Experimental design

To elucidate the presumed causal relationship between lipid metabolites and atherosclerosis, a two-sample Mendelian randomization (MR) approach was used. Single nucleotide polymorphisms (SNPs) were used as instrumental variables (IVs). This SNP-centered approach reflects the principle of a randomized controlled trial and helps identify the causal relationship between exposure factors (lipid metabolites) and atherosclerotic outcomes.

### Study design drawing


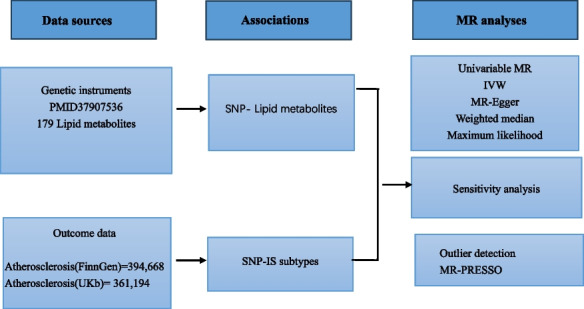
 
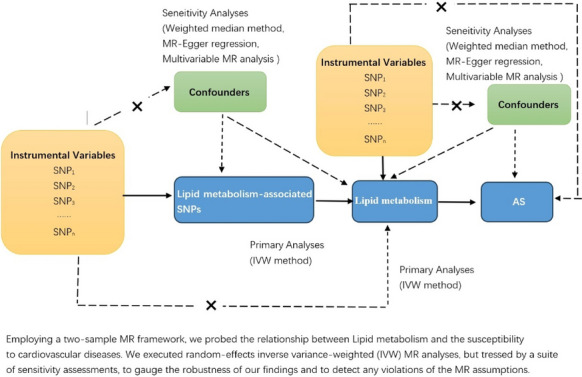


### Ethics and data use statement

Data were incorporated from previous studies that had been approved by the relevant institutional review board. Participants in the original study provided informed consent, so no further ethical review was required for this analysis. Nonetheless, we obtained an ethical statement from the institutional review board and ensured informed consent from all participants involved in the study.

### Genetic instrument variants for exposure

Our analyses used 179 SNPs associated with lipid metabolite levels from the PMID37907536. SNPs were selected if *p* < 5 × 10^−8, and SNPs were excluded if Maf < 0.01 and F > 10.

### GWAS Summary Data for Atherosclerosis

We retrieved data from the FinnGen dataset (https://gwas.mrcieu.ac.uk/datasets/finn-R10-I9/) and the UKb dataset (https://gwas.mrcieu.ac.uk/datasets/ukb-d-I9/) using the keyword "Atherosclerosis". The FinnGen dataset included 161 exposures with SNPs ≥ 1, and the combined GWAS summary data for AS could be found at finngen_R10_I9_CORATHER, including 51,589 cases and 343,079 controls. The UKb dataset included 162 exposures with SNPs ≥ 1, and the combined GWAS summary data for atherosclerosis could be found at ukb-d-I9_CORATHER, including 14,334 cases and 346,860 controls. Diagnostic criteria for atherosclerosis were based on a comprehensive assessment of glucose and lipids, calcium deposition on x-ray, and atherosclerotic plaques confirmed by arteriography, or Doppler ultrasound. Through IVW analysis (SNPs > 1) and Wald Ratio (SNP = 1) methods, we explored the potential causal relationship between the levels of each lipid metabolite and atherosclerosis. Also, each genetic marker surpassed the threshold for genome-wide significance (*P* < 0.001), indicating robust instrument strength (F-statistic > 10).

### Screening of IVs

The process of screening SNPs was meticulous attention to detail. Initially, SNPs closely associated with atherosclerosis were selected, with a genome-wide significance threshold of *P* < 5 × 10^−8. To ensure SNP independence and minimize the influence of linkage disequilibrium (LD), a stringent r^2^ of 0.01 was implemented within a 10,000 kb range. This step was essential to reduce the potential bias from LD. Additionally, the relationship between (IVs and the exposure was quantified using the F-statistic for each SNP, with IVs exhibiting an F-statistic > 10 indicating unbiased estimates.

### Statistical analysis

The primary method was the inverse variance-weighting (IVW) approach, which was predicated on the assumption that all SNPs were valid IVs, thus providing the most accurate estimates. If any SNP did not conform to the IV assumption, a modified version known as the random-effects IVW method was utilized. This method adjusted each estimate based on its standard error, thereby accounting for potential heterogeneity. The weighted median approach required that at least 50% of SNPs are valid to maintain the integrity of the IV assumption. The SNPs were ranked according to their weights and the experimental outcomes were examined to determine the median of the corresponding distribution. Furthermore, the MR-Egger regression, independent of the absence of pleiotropic effects, was used to derive an effect estimate. The intercept from the MR-Egger regression was used to evaluate the pleiotropic effect, with a non-significant deviation from zero indicating no directional pleiotropic bias.

### Sensitivity analysis

The random-effects IVW method was the cornerstone of our analysis of the causal links between atherosclerosis and lipid metabolites. This method synthesized the Wald ratio estimates for each SNP to obtain a causal estimate for each risk factor, yielding reliable estimates in the absence of pleiotropy. Sensitivity analyses were conducted to confirm the associations. The weighted median method was employed, which required only half of SNPs to be valid instruments, and the MR-Egger approach was utilized to accommodate a non-zero intercept, indicating pleiotropy. The MR-PRESSO test was employed to identify potential outliers, with adjustments made by excluding such SNPs. If, The IVW-MR estimates were considered robust if the adjusted effect was consistent with the uncorrected effect. However, in case of significant discrepancies, the adjusted effects should be prioritized, as they may be less biased and better reflect the true relationship.

A two-stage MR analysis was performed to evaluate the mediating effects. The first stage used a genetic instrument of the lipid metabolites to estimate the causal effect of the exposure on the mediator. The second stage employed genetic instruments of the mediator to ascertain the causal effect on the risk of atherosclerosis.

The causal effects of lipid metabolites on the risk of atherosclerosis were described using odds ratios (ORs), beta coefficients (β), and 95% confidence intervals (CIs). MR and sensitivity analyses were performed using R software (version 4.2.1) and the "TwoSampleMR" package (version 0.5.6). In the univariate MR analyses, a *P*-value of < 3.11 × 10^−4 (FinnGen dataset) or a *P*-value of < 3.09 × 10^−4 (UKb dataset) (adjusted for multiple comparisons as 0.05 divided by the number of exposures and outcomes) implied a statistically significant causal relationship.

## Results

### MR Analysis: the role of lipid metabolites in atherosclerosis (FinnGen dataset)

**Fig. 1:**
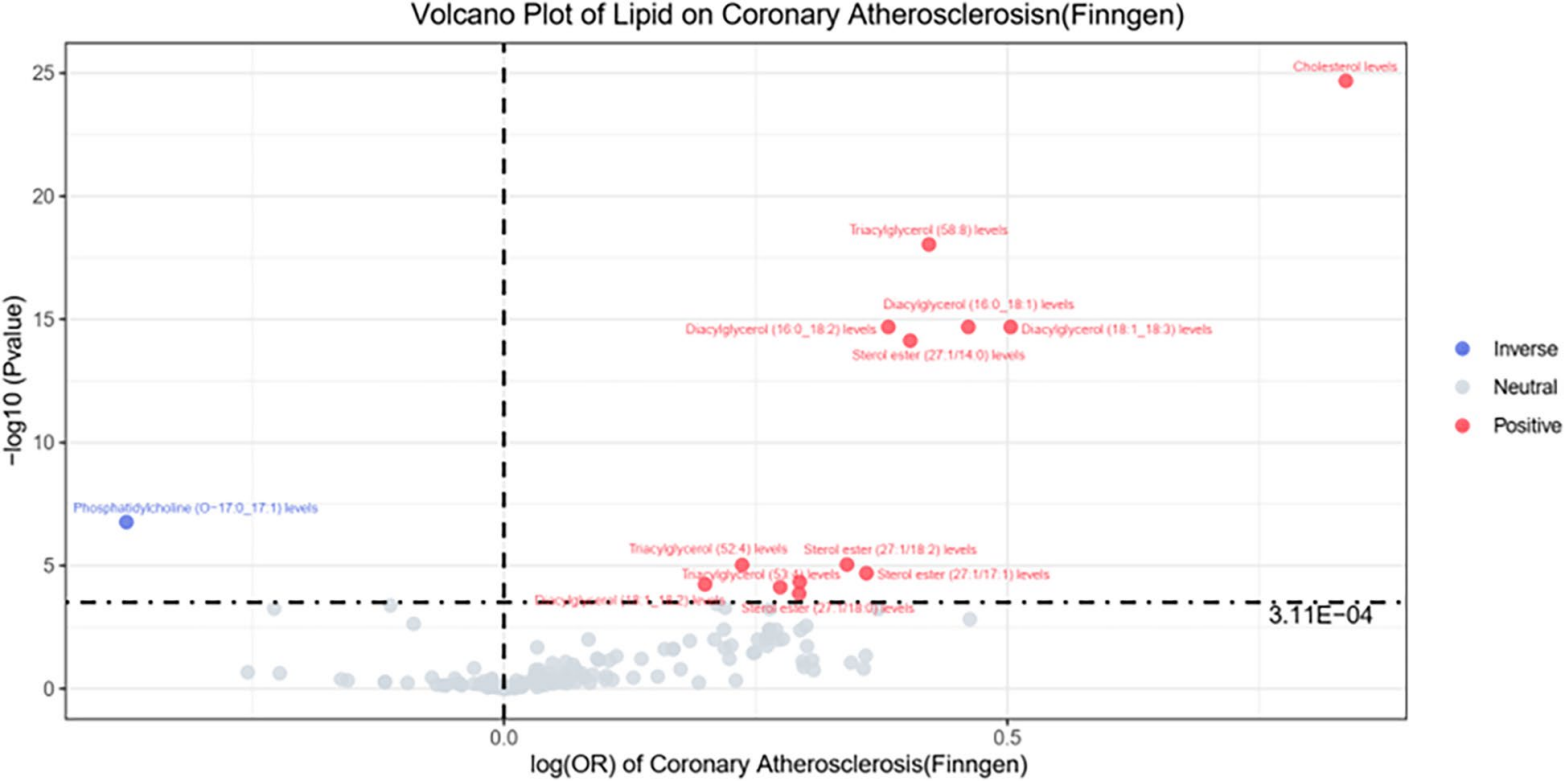
Volcano Plot of Lipid on Coronary Atherosclerosis (Finngen dataset). Note: The horizontal coordinate represents the relative risk of atherosclerosis and the vertical coordinate represents the *p*-value

**Fig. 2:**
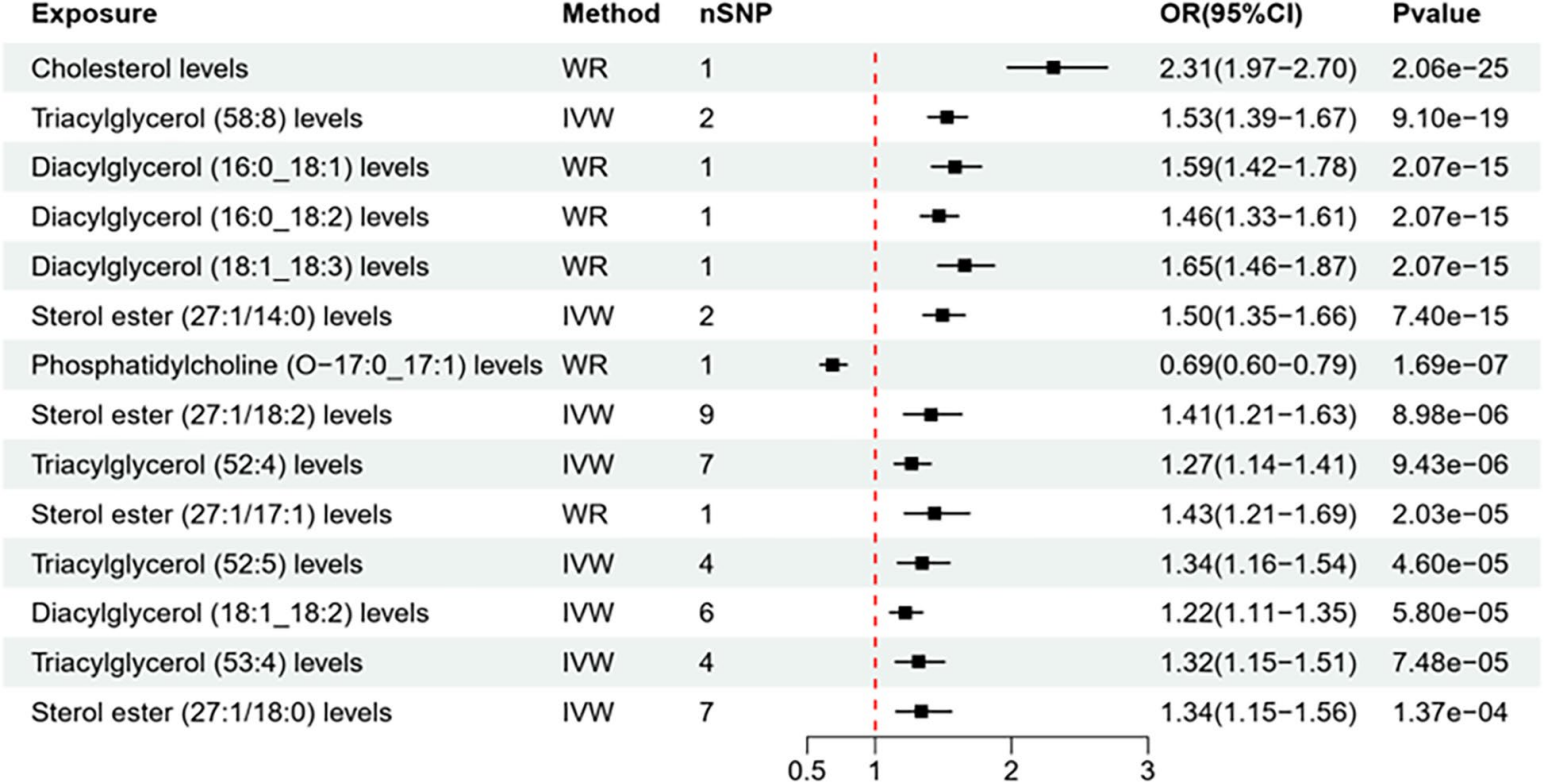
Forest plot of lipid metabolites on atherosclerosis risk (Finngen dataset).Note: Images from left to right the first column is Exposure, the second column is Method, the third column represents nSNP, the fourth column is OR (95% CI) value, and the last column is *P* value

Fourteen out of 161 exposures with a *P* < 0.001 were found. By applying IVW analyses and weighted median methods, we explored potential causal associations between lipid metabolite levels and atherosclerosis. In the Finn database, the IVW methods unveiled significant associations, implying that elevated levels of lipid metabolites may increase the risk of atherosclerosis. The influence of each lipid metabolite on the risk of atherosclerosis is represented in the volcano plot (Fig. 1) and detailed data can be found in Table 1. The *P*-value for exposure to 14 lipid metabolites on the risk of atherosclerosis was less than 0.001, as shown by a forest plot (Fig. 2).

**Table 1:** Finngen dataset of lipid metabolites on atherosclerosis risk

No	Exposure	Method	nSNP	OR(95%CI)	*P* value
1	Cholesterol levels	WR	1	2.31(1.97–2.70)	2.06E-25
2	Triacylglycerol (58:8) levels	IVW	2	1.53(1.39–1.67)	9.10E-19
3	Diacylglycerol (16:0_18:1) levels	WR	1	1.59(1.42–1.78)	2.07E-15
4	Diacylglycerol (16:0_18:2) levels	WR	1	1.46(1.33–1.61)	2.07E-15
5	Diacylglycerol (18:1_18:3) levels	WR	1	1.65(1.46–1.87)	2.07E-15
6	Sterol ester (27:1/14:0) levels	IVW	2	1.50(1.35–1.66)	7.40E-15
7	Phosphatidylcholine (O-17:0_17:1) levels	WR	1	0.69(0.60–0.79)	1.69E-07
8	Sterol ester (27:1/18:2) levels	IVW	9	1.41(1.21–1.63)	8.98E-06
9	Triacylglycerol (52:4) levels	IVW	7	1.27(1.14–1.41)	9.43E-06
10	Sterol ester (27:1/17:1) levels	WR	1	1.43(1.21–1.69)	2.03E-05
11	Triacylglycerol (52:5) levels	IVW	4	1.34(1.16–1.54)	4.60E-05
12	Diacylglycerol (18:1_18:2) levels	IVW	6	1.22(1.11–1.35)	5.80E-05
13	Triacylglycerol (53:4) levels	IVW	4	1.32(1.15–1.51)	7.48E-05
14	Sterol ester (27:1/18:0) levels	IVW	7	1.34(1.15–1.56)	0.000136624
15	Triacylglycerol (52:3) levels	IVW	6	1.24(1.10–1.39)	0.000367859
16	Phosphatidylcholine (16:0_20:3) levels	IVW	2	0.89(0.84–0.95)	0.00041228
17	Triacylglycerol (54:4) levels	IVW	6	1.25(1.10–1.41)	0.000512616
18	Phosphatidylcholine (O-18:0_16:1) levels	WR	1	0.80(0.70–0.91)	0.000555411
19	Triacylglycerol (51:3) levels	IVW	6	1.30(1.12–1.51)	0.000580395
20	Triacylglycerol (50:1) levels	IVW	3	1.45(1.17–1.79)	0.00058479
21	Sterol ester (27:1/18:1) levels	IVW	6	1.59(1.19–2.11)	0.00150153
22	Phosphatidylcholine (18:0_20:3) levels	IVW	3	0.91(0.86–0.97)	0.00231212
23	Triacylglycerol (53:2) levels	IVW	2	1.35(1.11–1.64)	0.002801806
24	Sterol ester (27:1/16:0) levels	IVW	9	1.30(1.09–1.56)	0.003863431
25	Sterol ester (27:1/20:2) levels	IVW	3	1.24(1.07–1.44)	0.003953403
26	Triacylglycerol (50:3) levels	IVW	3	1.31(1.09–1.58)	0.004007562
27	Triacylglycerol (52:2) levels	IVW	4	1.34(1.10–1.64)	0.004200314
28	Triacylglycerol (54:5) levels	IVW	4	1.30(1.09–1.56)	0.004384377
29	Sphingomyelin (d34:1) levels	IVW	7	1.30(1.08–1.57)	0.006416098
30	Triacylglycerol (51:4) levels	IVW	2	1.32(1.07–1.63)	0.009523203
31	Triacylglycerol (50:4) levels	IVW	3	1.30(1.06- 1.58)	0.009925336
32	Triacylglycerol (53:3) levels	IVW	6	1.23(1.05–1.45)	0.009998098
33	Phosphatidylcholine (O-16:2_18:0) levels	IVW	2	1.09(1.02–1.16)	0.0100131
34	Triacylglycerol (56:7) levels	IVW	5	1.29(1.06–1.56)	0.010074157
35	Triacylglycerol (50:5) levels	IVW	3	1.31(1.07–1.62)	0.010359191
36	Diacylglycerol (18:1_18:1) levels	IVW	5	1.20(1.04–1.39)	0.011355585
37	Triacylglycerol (54:3) levels	IVW	4	1.25(1.04–1.51)	0.017438794
38	Sphingomyelin (d42:2) levels	IVW	9	1.30(1.05–1.61)	0.018204147
39	Triacylglycerol (51:2) levels	IVW	2	1.35(1.05–1.74)	0.018830444
40	Phosphatidylethanolamine (16:0_20:4) levels	IVW	5	1.03(1.01–1.06)	0.020840435
41	Triacylglycerol (52:6) levels	IVW	4	1.25(1.03–1.50)	0.022170992
42	Phosphatidylcholine (16:0_16:1) levels	WR	1	1.18(1.02–1.37)	0.024598158
43	Phosphatidylcholine (16:1_18:0) levels	WR	1	1.18(1.02–1.37)	0.024598158
44	Triacylglycerol (46:2) levels	WR	1	1.17(1.02–1.35)	0.024598158
45	Triacylglycerol (54:6) levels	IVW	3	1.28(1.02–1.61)	0.032887895
46	Triacylglycerol (54:7) levels	IVW	4	1.28(1.02–1.61)	0.035398945
47	Diacylglycerol (16:1_18:1) levels	IVW	2	1.43(1.01–2.04)	0.045686665
48	Phosphatidylcholine (16:0_18:1) levels	IVW	2	1.12(1.00–1.25)	0.047744613
49	Phosphatidylethanolamine (O-18:2_18:2) levels	WR	1	1.10(1.00–1.21)	0.05910026
50	Triacylglycerol (56:8) levels	IVW	5	1.25(0.99–1.58)	0.060719079
51	Sphingomyelin (d36:1) levels	IVW	9	1.15(0.99–1.32)	0.061033083
52	Phosphatidylcholine (16:0_0:0) levels	IVW	2	1.10(0.99–1.22)	0.069255821
53	Sterol ester (27:1/17:0) levels	IVW	2	1.36(0.98–1.89)	0.069346504
54	Sterol ester (27:1/18:3) levels	IVW	5	1.11(0.99–1.24)	0.071317159
55	Triacylglycerol (50:2) levels	IVW	2	1.35(0.97–1.87)	0.077572305
56	Phosphatidylethanolamine (18:1_18:1) levels	IVW	7	1.06(0.99–1.14)	0.080867196
57	Triacylglycerol (49:2) levels	IVW	2	1.41(0.95–2.10)	0.087558732
58	Phosphatidylethanolamine (16:0_18:2) levels	IVW	6	1.05(0.99–1.11)	0.091570989
59	Phosphatidylcholine (O-16:1_16:0) levels	WR	1	1.07(0.99–1.16)	0.106235712
60	Triacylglycerol (48:3) levels	IVW	2	1.35(0.91–1.98)	0.131224652
61	Sphingomyelin (d32:1) levels	IVW	3	0.97(0.93–1.01)	0.147871677
62	Triacylglycerol (48:1) levels	IVW	2	1.43(0.88–2.33)	0.152630194
63	Triacylglycerol (56:4) levels	IVW	3	1.19(0.93–1.52)	0.162150321
64	Phosphatidylinositol (16:0_18:1) levels	IVW	2	1.07(0.97–1.19)	0.167164428
65	Phosphatidylinositol (16:0_18:2) levels	IVW	3	1.04(0.99–1.09)	0.169603646
66	Sphingomyelin (d34:0) levels	IVW	3	1.03(0.99–1.08)	0.17209998
67	Triacylglycerol (48:2) levels	IVW	2	1.36(0.87–2.12)	0.175396599
68	Phosphatidylcholine (O-16:0_20:3) levels	IVW	3	0.78(0.52–1.16)	0.21472013
69	Phosphatidylinositol (18:0_18:2) levels	IVW	6	1.06(0.96–1.16)	0.228379302
70	Phosphatidylethanolamine (18:0_18:2) levels	IVW	10	1.05(0.97–1.13)	0.23431811
71	Phosphatidylcholine (O-16:1_18:1) levels	IVW	3	0.80(0.55–1.16)	0.236096566
72	Phosphatidylinositol (18:0_18:1) levels	IVW	6	1.08(0.95–1.23)	0.240569804
73	Sphingomyelin (d40:1) levels	IVW	7	1.09(0.94–1.28)	0.263520485
74	Phosphatidylinositol (18:1_18:2) levels	IVW	3	1.04(0.97–1.11)	0.277806447
75	Sphingomyelin (d38:1) levels	IVW	10	1.06(0.95–1.19)	0.287128535
76	Phosphatidylinositol (18:1_18:1) levels	IVW	5	1.11(0.91–1.34)	0.298008439
77	Phosphatidylethanolamine (18:0_20:4) levels	IVW	8	1.03(0.97–1.09)	0.308760301
78	Triacylglycerol (56:5) levels	IVW	4	1.16(0.86- 1.58)	0.320966903
79	Sphingomyelin (d38:2) levels	IVW	6	1.04(0.97–1.11)	0.327053359
80	Phosphatidylcholine (O-16:1_20:3) levels	IVW	3	0.93(0.80–1.08)	0.353709809
81	Sphingomyelin (d34:2) levels	IVW	5	1.14(0.86–1.49)	0.357238502
82	Phosphatidylcholine (18:2_20:4) levels	IVW	4	1.08(0.91–1.29)	0.3657876
83	Phosphatidylcholine (18:0_18:1) levels	IVW	2	0.95(0.85–1.06)	0.381266322
84	Phosphatidylcholine (20:4_0:0) levels	IVW	4	0.99(0.96–1.02)	0.388590869
85	Phosphatidylcholine (O-16:1_18:2) levels	IVW	2	0.85(0.58–1.25)	0.41236352
86	Ceramide (d42:1) levels	IVW	3	1.07(0.91–1.25)	0.419941618
87	Phosphatidylcholine (17:0_18:2) levels	IVW	3	1.11(0.85–1.46)	0.431331784
88	Ceramide (d42:2) levels	IVW	8	1.04(0.94–1.16)	0.431698515
89	Phosphatidylcholine (O-18:2_20:4) levels	IVW	2	1.03(0.96–1.11)	0.435446807
90	Phosphatidylcholine (16:0_20:4) levels	IVW	3	1.11(0.86–1.43)	0.436989488
91	Phosphatidylinositol (16:0_20:4) levels	IVW	3	1.03(0.95–1.11)	0.450094084
92	Ceramide (d40:1) levels	IVW	6	1.05(0.93–1.19)	0.452391397
93	Phosphatidylcholine (18:2_18:2) levels	IVW	3	1.04(0.93–1.17)	0.453618054
94	Phosphatidylcholine (16:0_20:2) levels	IVW	6	1.03(0.95–1.12)	0.458230865
95	Phosphatidylinositol (18:1_20:4) levels	IVW	2	1.26(0.68–2.32)	0.461168643
96	Phosphatidylethanolamine (18:0_0:0) levels	IVW	2	1.04(0.94–1.14)	0.462048634
97	Phosphatidylcholine (O-16:0_16:1) levels	IVW	2	0.86(0.57–1.30)	0.463523251
98	Phosphatidylcholine (O-16:1_20:4) levels	IVW	3	1.01(0.98–1.05)	0.465212122
99	Ceramide (d40:2) levels	IVW	2	0.98(0.92–1.04)	0.495150006
100	Sterol ester (27:1/20:5) levels	IVW	3	0.99(0.95–1.02)	0.496189805
101	Sterol ester (27:1/20:4) levels	IVW	8	1.04(0.92–1.19)	0.50390227
102	Phosphatidylinositol (18:0_20:4) levels	IVW	7	1.04(0.93–1.15)	0.508039155
103	Triacylglycerol (56:6) levels	IVW	7	1.07(0.86–1.34)	0.523177997
104	Phosphatidylcholine (O-18:2_18:2) levels	IVW	2	0.89(0.62–1.28)	0.528096266
105	Phosphatidylcholine (O-18:2_16:0) levels	IVW	2	0.89(0.61–1.29)	0.532607275
106	Phosphatidylcholine (14:0_18:2) levels	IVW	3	1.02(0.96–1.08)	0.544368134
107	Sphingomyelin (d40:2) levels	IVW	7	1.06(0.88–1.27)	0.549347207
108	Sterol ester (27:1/22:6) levels	IVW	2	1.21(0.63–2.34)	0.563765951
109	Triacylglycerol (58:7) levels	IVW	6	1.09(0.81–1.46)	0.569949028
110	Phosphatidylcholine (O-16:0_18:2) levels	IVW	2	0.91(0.65–1.27)	0.575980737
111	Phosphatidylcholine (18:1_18:1) levels	IVW	4	0.95(0.81–1.13)	0.580667249
112	Phosphatidylcholine (16:0_22:5) levels	IVW	4	0.99(0.96–1.02)	0.58462494
113	Phosphatidylcholine (18:0_18:2) levels	IVW	5	1.06(0.85–1.34)	0.595050799
114	Phosphatidylcholine (14:0_18:1) levels	IVW	2	1.05(0.87–1.27)	0.614292473
115	Phosphatidylcholine (18:1_0:0) levels	WR	1	1.04(0.90–1.19)	0.616247984
116	Phosphatidylcholine (O-18:1_20:4) levels	IVW	4	0.96(0.80–1.14)	0.624365879
117	Phosphatidylcholine (16:0_22:4) levels	IVW	2	0.99(0.94–1.04)	0.630073271
118	Phosphatidylcholine (17:0_18:1) levels	WR	1	1.03(0.90–1.19)	0.630384458
119	Phosphatidylcholine (16:1_18:1) levels	IVW	5	0.97(0.86–1.10)	0.635679846
120	Phosphatidylcholine (16:0_18:2) levels	IVW	7	1.03(0.90–1.19)	0.636123
121	Phosphatidylethanolamine (O-16:1_20:4) levels	IVW	2	0.99(0.93–1.05)	0.652836475
122	Phosphatidylcholine (O-16:0_20:4) levels	IVW	3	0.99(0.95–1.03)	0.6608036
123	Phosphatidylcholine (O-18:1_20:3) levels	IVW	2	0.98(0.87–1.09)	0.676095676
124	Phosphatidylcholine (16:0_18:3) levels	IVW	3	0.94( 0.68- 1.29)	0.687458624
125	Phosphatidylcholine (18:1_20:4) levels	IVW	6	1.02(0.93–1.11)	0.688321371
126	Sterol ester (27:1/20:3) levels	IVW	6	1.04(0.83–1.30)	0.721893127
127	Phosphatidylcholine (18:0_20:4) levels	IVW	6	1.01(0.97–1.05)	0.727556475
128	Phosphatidylcholine (O-18:1_16:0) levels	IVW	3	0.94(0.67–1.33)	0.730309613
129	Phosphatidylcholine (18:1_20:3) levels	IVW	3	0.96(0.75–1.22)	0.734376192
130	Phosphatidylcholine (O-16:0_18:1) levels	IVW	3	0.94(0.67- 1.34)	0.745359543
131	Phosphatidylethanolamine (O-18:1_20:4) levels	IVW	2	0.99(0.94–1.05)	0.752732493
132	Phosphatidylcholine (18:0_20:5) levels	IVW	2	0.99(0.96–1.03)	0.767554587
133	Sphingomyelin (d36:2) levels	IVW	4	1.02(0.91–1.13)	0.774221869
134	Phosphatidylethanolamine (18:2_0:0) levels	IVW	2	1.01(0.93–1.11)	0.778881979
135	Phosphatidylcholine (16:0_20:1) levels	WR	1	1.02(0.89–1.16)	0.798165075
136	Phosphatidylethanolamine (O-18:2_20:4) levels	IVW	2	0.99(0.92–1.07)	0.808163188
137	Phosphatidylcholine (18:0_22:5) levels	IVW	2	1.01(0.91–1.13)	0.816535299
138	Phosphatidylcholine (16:1_18:2) levels	IVW	8	0.99(0.90–1.09)	0.826920769
139	Phosphatidylcholine (15:0_18:2) levels	IVW	6	1.02(0.86–1.20)	0.830942874
140	Phosphatidylcholine (O-18:2_18:1) levels	WR	1	1.01(0.91–1.12)	0.844814769
141	Phosphatidylcholine (18:0_18:3) levels	WR	1	0.99(0.85–1.14)	0.849376935
142	Phosphatidylethanolamine (O-16:1_18:2) levels	WR	1	1.01(0.91–1.12)	0.855722939
143	Sterol ester (27:1/16:1) levels	IVW	3	1.03(0.72- 1.48)	0.858180553
144	Phosphatidylcholine (18:1_20:2) levels	IVW	3	1.00(0.95–1.06)	0.876467317
145	Phosphatidylcholine (18:0_22:6) levels	IVW	2	0.99(0.92–1.08)	0.88254596
146	Phosphatidylethanolamine (O-18:1_18:2) levels	WR	1	1.01(0.93–1.09)	0.887407734
147	Phosphatidylcholine (O-18:1_18:2) levels	WR	1	1.00(0.94–1.07)	0.888884555
148	Phosphatidylcholine (16:0_16:0) levels	IVW	4	0.98(0.77–1.26)	0.899936549
149	Phosphatidylcholine (18:1_18:2) levels	IVW	5	0.99(0.89–1.11)	0.905095502
150	Phosphatidylinositol (18:0_20:3) levels	IVW	5	1.01(0.88–1.16)	0.91701762
151	Phosphatidylcholine (18:0_20:2) levels	WR	1	1.00(0.95–1.06)	0.918093888
152	Phosphatidylcholine (16:1_20:4) levels	WR	1	1.00(0.95–1.05)	0.933573628
153	Phosphatidylcholine (18:2_0:0) levels	IVW	3	1.00(0.93–1.09)	0.935282319
154	Phosphatidylcholine (16:0_20:5) levels	WR	1	1.00(0.96–1.04)	0.935910484
155	Phosphatidylcholine (17:0_20:4) levels	IVW	5	1.00(0.94–1.07)	0.942444677
156	Phosphatidylcholine (16:0_22:6) levels	WR	1	1.00(0.89–1.13)	0.943754227
157	Phosphatidylcholine (16:0_18:0) levels	WR	1	1.00(0.90–1.11)	0.94496326
158	Triacylglycerol (56:3) levels	IVW	4	1.01(0.65- 1.57)	0.960799264
159	Phosphatidylcholine (18:2_20:3) levels	WR	1	1.00(0.93–1.08)	0.983858771
160	Phosphatidylcholine (O-16:0_22:5) levels	WR	1	1.00(0.91–1.10)	0.983858771
161	Phosphatidylcholine (O-18:0_20:4) levels	IVW	2	1.00(0.95–1.05)	0.997478043

### MR Analysis: the role of lipid metabolites in atherosclerosis (UKb dataset)

**Fig. 3:**
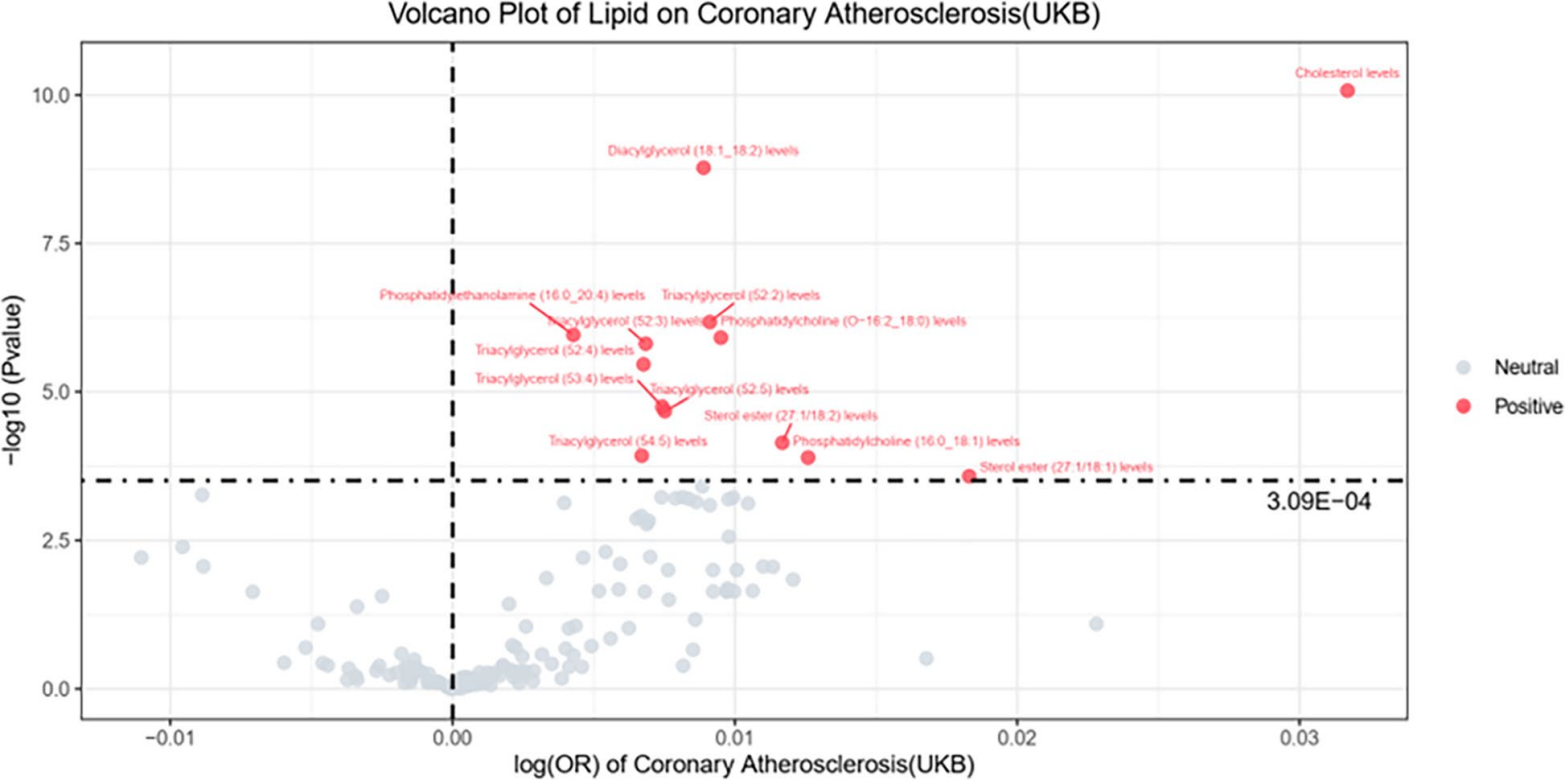
Volcano Plot of Lipid on Coronary Atherosclerosis (UKB dataset). Note: The horizontal coordinate represents the relative risk of atherosclerosis and the vertical coordinate represents the p-value

**Fig. 4:**
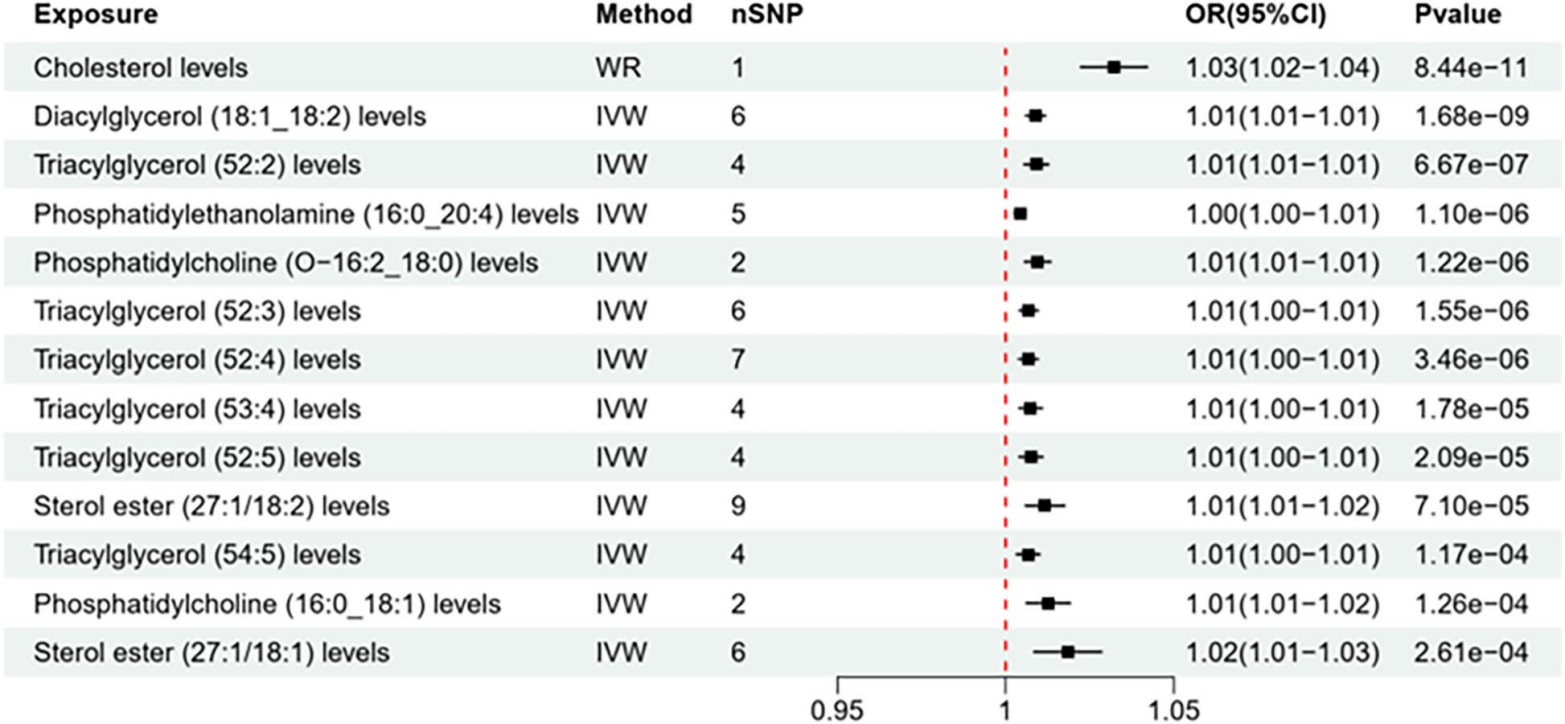
Forest plot of lipid metabolites on atherosclerosis risk (UKB dataset). Note: Images from left to right the first column is Exposure, the second column is Method, the third column represents nSNP, the fourth column is OR (95% CI) value, and the last column is P value

Thirteen out of 162 Exposures were found to have a *P* < 0.001. By applying (IVW analyses and weighted median methods, we explored potential causal associations between lipid metabolite levels and atherosclerosis. In the UKb database, the IVW methods revealed notable associations, implying that elevated levels of lipid metabolites may increase the risk of atherosclerosis. The influence of each lipid metabolite on atherosclerosis risk is graphically represented in the volcano plot (Fig. 3) and detailed data can be found in Table 2. The *P* value for exposure to 13 lipid metabolites for atherosclerosis risk was ≤ 0.001, as represented by a forest plot (Fig. 4).

**Table 2:** UKB dataset of lipid metabolites on atherosclerosis risk

No	Exposure	Method	nSNP	OR(95%CI)	Pvalue
1	Cholesterol levels	WR	1	1.03(1.02–1.04)	8.44E-11
2	Diacylglycerol (18:1_18:2) levels	IVW	6	1.01(1.01–1.01)	1.68E-09
3	Triacylglycerol (52:2) levels	IVW	4	1.01(1.01–1.01)	6.67E-07
4	Phosphatidylethanolamine (16:0_20:4) levels	IVW	5	1.00(1.00–1.01)	1.10E-06
5	Phosphatidylcholine (O-16:2_18:0) levels	IVW	2	1.01(1.01–1.01)	1.22E-06
6	Triacylglycerol (52:3) levels	IVW	6	1.01(1.00–1.01)	1.55E-06
7	Triacylglycerol (52:4) levels	IVW	7	1.01(1.00–1.01)	3.46E-06
8	Triacylglycerol (53:4) levels	IVW	4	1.01(1.00–1.01)	1.78E-05
9	Triacylglycerol (52:5) levels	IVW	4	1.01(1.00–1.01)	2.09E-05
10	Sterol ester (27:1/18:2) levels	IVW	9	1.01(1.01–1.02)	7.10E-05
11	Triacylglycerol (54:5) levels	IVW	4	1.01(1.00–1.01)	0.000117
12	Phosphatidylcholine (16:0_18:1) levels	IVW	2	1.01(1.01–1.02)	0.000126
13	Sterol ester (27:1/18:1) levels	IVW	6	1.02(1.01–1.03)	0.000261
14	Triacylglycerol (50:1) levels	IVW	3	1.01(1.00–1.01)	0.000386
15	Phosphatidylcholine (O-16:0_16:1) levels	IVW	2	0.99(0.99–1.00)	0.000537
16	Triacylglycerol (51:2) levels	IVW	2	1.01(1.00–1.01)	0.000584
17	Triacylglycerol (51:4) levels	IVW	2	1.01(1.00–1.01)	0.000591
18	Diacylglycerol (16:1_18:1) levels	IVW	2	1.01(1.00–1.02)	0.000595
19	Triacylglycerol (53:2) levels	IVW	2	1.01(1.00–1.01)	0.000614
20	Triacylglycerol (50:2) levels	IVW	2	1.01(1.00–1.01)	0.000627
21	Triacylglycerol (49:2) levels	IVW	2	1.01(1.00–1.02)	0.00064
22	Triacylglycerol (48:3) levels	IVW	2	1.01(1.00–1.01)	0.000708
23	Phosphatidylethanolamine (18:1_18:1) levels	IVW	7	1.00(1.00–1.01)	0.000735
24	Triacylglycerol (48:1) levels	IVW	2	1.01(1.00–1.02)	0.00075
25	Triacylglycerol (48:2) levels	IVW	2	1.01(1.00–1.01)	0.000801
26	Triacylglycerol (50:3) levels	IVW	3	1.01(1.00–1.01)	0.001218
27	Triacylglycerol (50:4) levels	IVW	3	1.01(1.00–1.01)	0.001361
28	Triacylglycerol (54:6) levels	IVW	3	1.01(1.00–1.01)	0.001471
29	Triacylglycerol (50:5) levels	IVW	3	1.01(1.00–1.01)	0.001675
30	Sterol ester (27:1/16:0) levels	IVW	9	1.01(1.00–1.02)	0.002737
31	Phosphatidylcholine (O-18:0_16:1) levels	WR	1	0.99(0.98–1.00)	0.00404
32	Triacylglycerol (52:6) levels	IVW	4	1.01(1.00–1.01)	0.004911
33	Triacylglycerol (51:3) levels	IVW	6	1.01(1.00–1.01)	0.005971
34	Phosphatidylcholine (O-17:0_17:1) levels	WR	1	0.99(0.98–1.00)	0.006106
35	Diacylglycerol (18:1_18:1) levels	IVW	5	1.00(1.00–1.01)	0.006147
36	Triacylglycerol (54:7) levels	IVW	4	1.01(1.00–1.01)	0.007843
37	Phosphatidylcholine (18:0_18:1) levels	IVW	2	0.99(0.98–1.00)	0.008594
38	Phosphatidylcholine (17:0_18:1) levels	WR	1	1.01(1.00–1.02)	0.008633
39	Sphingomyelin (d34:1) levels	IVW	7	1.01(1.00–1.02)	0.008781
40	Diacylglycerol (16:0_18:2) levels	WR	1	1.01(1.00–1.01)	0.009928
41	Diacylglycerol (16:0_18:1) levels	WR	1	1.01(1.00–1.02)	0.009928
42	Diacylglycerol (18:1_18:3) levels	WR	1	1.01(1.00–1.02)	0.009928
43	Phosphatidylethanolamine (16:0_18:2) levels	IVW	6	1.00(1.00–1.01)	0.01361
44	Sphingomyelin (d42:2) levels	IVW	9	1.01(1.00–1.02)	0.014355
45	Sterol ester (27:1/18:0) levels	IVW	7	1.01(1.00–1.02)	0.020294
46	Triacylglycerol (53:3) levels	IVW	6	1.01(1.00–1.01)	0.021034
47	Sterol ester (27:1/20:2) levels	IVW	3	1.01(1.00–1.02)	0.022305
48	Phosphatidylinositol (16:0_20:4) levels	IVW	3	1.01(1.00–1.01)	0.022424
49	Phosphatidylcholine (14:0_18:1) levels	WR	1	1.01(1.00–1.02)	0.022874
50	Phosphatidylcholine (16:0_16:1) levels	WR	1	1.01(1.00–1.02)	0.022874
51	Phosphatidylcholine (16:1_18:0) levels	WR	1	1.01(1.00–1.02)	0.022874
52	Triacylglycerol (46:2) levels	WR	1	1.01(1.00–1.02)	0.022874
53	Triacylglycerol (56:7) levels	IVW	5	1.01(1.00–1.01)	0.0231
54	Sterol ester (27:1/16:1) levels	IVW	3	0.99(0.99–1.00)	0.023108
55	Sphingomyelin (d32:1) levels	IVW	3	1.00(1.00–1.00)	0.027294
56	Triacylglycerol (58:8) levels	IVW	2	1.01(1.00–1.01)	0.031439
57	Phosphatidylinositol (18:0_20:4) levels	IVW	7	1.00(1.00–1.00)	0.037175
58	Ceramide (d40:2) levels	IVW	2	1.00(0.99–1.00)	0.041006
59	Sphingomyelin (d34:2) levels	IVW	5	1.01(1.00–1.02)	0.067901
60	Sterol ester (27:1/14:0) levels	IVW	2	1.02(1.00–1.05)	0.080481
61	Phosphatidylcholine (18:2_0:0) levels	IVW	3	1.00(0.99–1.00)	0.080643
62	Triacylglycerol (54:4) levels	IVW	6	1.00(1.00–1.01)	0.086445
63	Phosphatidylethanolamine (18:0_18:2) levels	IVW	10	1.00(1.00–1.01)	0.088805
64	Sphingomyelin (d40:1) levels	IVW	7	1.01(1.00–1.01)	0.09434
65	Phosphatidylcholine (O-16:1_16:0) levels	WR	1	1.00(1.00–1.01)	0.095633
66	Triacylglycerol (56:8) levels	IVW	5	1.01(1.00–1.01)	0.141722
67	Sphingomyelin (d34:0) levels	IVW	3	1.00(1.00–1.01)	0.184354
68	Sterol ester (27:1/18:3) levels	IVW	5	1.00(1.00–1.01)	0.18971
69	Phosphatidylethanolamine (18:0_20:4) levels	IVW	8	1.00(1.00–1.01)	0.200899
70	Phosphatidylcholine (18:1_0:0) levels	WR	1	0.99(0.99–1.00)	0.201497
71	Triacylglycerol (54:3) levels	IVW	4	1.00(1.00–1.01)	0.209889
72	Phosphatidylinositol (18:1_20:4) levels	IVW	2	1.01(0.99–1.02)	0.21837
73	Phosphatidylcholine (18:0_20:3) levels	IVW	3	1.00(1.00–1.00)	0.255277
74	Phosphatidylcholine (16:0_20:2) levels	IVW	6	1.00(1.00–1.01)	0.262575
75	Triacylglycerol (56:5) levels	IVW	4	1.00(1.00–1.01)	0.272391
76	Phosphatidylcholine (O-18:2_20:4) levels	IVW	2	1.00(1.00–1.01)	0.283406
77	Sterol ester (27:1/17:0) levels	IVW	2	1.02(0.98–1.05)	0.307198
78	Ceramide (d40:1) levels	IVW	6	1.00(1.00–1.00)	0.320548
79	Phosphatidylcholine (O-16:0_20:3) levels	IVW	3	0.99(0.98–1.01)	0.362053
80	Phosphatidylcholine (O-18:2_16:0) levels	IVW	2	1.00(0.99–1.01)	0.368579
81	Phosphatidylethanolamine (O-18:2_18:2) levels	WR	1	1.00(1.00–1.01)	0.378032
82	Phosphatidylcholine (O-16:1_20:4) levels	IVW	3	1.00(1.00–1.01)	0.398009
83	Phosphatidylcholine (O-18:2_18:2) levels	IVW	2	1.00(0.99–1.01)	0.402248
84	Sterol ester (27:1/22:6) levels	IVW	2	1.01(0.99–1.03)	0.405987
85	Phosphatidylethanolamine (O-16:1_18:2) levels	WR	1	1.00(0.99–1.00)	0.407836
86	Ceramide (d42:2) levels	IVW	8	1.00(0.99–1.00)	0.413379
87	Sterol ester (27:1/20:4) levels	IVW	8	1.00(1.00–1.01)	0.423497
88	Phosphatidylcholine (16:0_16:0) levels	IVW	5	1.00(0.99–1.02)	0.424141
89	Phosphatidylcholine (18:2_18:2) levels	IVW	3	1.00(0.99–1.01)	0.426907
90	Phosphatidylcholine (16:0_20:3) levels	IVW	2	1.00(1.00–1.00)	0.433529
91	Phosphatidylcholine (O-16:0_18:2) levels	IVW	2	1.00(0.99–1.01)	0.453937
92	Ceramide (d42:1) levels	IVW	3	1.00(1.00–1.00)	0.476222
93	Phosphatidylcholine (18:2_20:3) levels	WR	1	1.00(0.99–1.00)	0.476919
94	Phosphatidylcholine (O-16:0_22:5) levels	WR	1	1.00(1.00–1.01)	0.476919
95	Phosphatidylcholine (18:1_20:3) levels	IVW	3	1.00(0.99–1.00)	0.493016
96	Sphingomyelin (d38:2) levels	IVW	5	1.00(0.99–1.01)	0.49352
97	Phosphatidylcholine (17:0_18:2) levels	IVW	3	1.00(0.99–1.01)	0.498044
98	Phosphatidylcholine (18:2_20:4) levels	IVW	4	1.00(1.00–1.01)	0.500011
99	Phosphatidylcholine (16:0_22:6) levels	WR	1	1.00(1.00–1.01)	0.504428
100	Triacylglycerol (56:4) levels	IVW	3	1.00(1.00–1.01)	0.523428
101	Phosphatidylinositol (18:1_18:1) levels	IVW	5	1.00(1.00–1.00)	0.525829
102	Phosphatidylcholine (O-18:1_20:3) levels	IVW	2	1.00(1.00–1.00)	0.527624
103	Phosphatidylinositol (18:0_18:2) levels	IVW	6	1.00(1.00–1.01)	0.530169
104	Phosphatidylcholine (16:0_18:0) levels	WR	1	1.00(0.99–1.00)	0.530577
105	Phosphatidylethanolamine (O-18:2_20:4) levels	IVW	2	1.00(1.00–1.01)	0.534978
106	Phosphatidylcholine (18:2_20:1) levels	WR	1	1.00(1.00–1.00)	0.55807
107	Phosphatidylcholine (16:0_18:2) levels	IVW	7	1.00(1.00–1.01)	0.579553
108	Phosphatidylcholine (16:0_20:1) levels	WR	1	1.00(0.99–1.01)	0.583773
109	Sphingomyelin (d36:1) levels	IVW	8	1.00(0.99–1.01)	0.584407
110	Phosphatidylcholine (18:0_22:5) levels	IVW	2	1.00(1.00–1.01)	0.598499
111	Triacylglycerol (56:3) levels	IVW	4	1.00(0.98–1.01)	0.59934
112	Phosphatidylcholine (17:0_20:4) levels	IVW	5	1.00(1.00–1.00)	0.636507
113	Phosphatidylinositol (16:0_18:1) levels	IVW	2	1.00(0.99–1.00)	0.636795
114	Phosphatidylcholine (18:0_20:5) levels	IVW	2	1.00(1.00–1.00)	0.639059
115	Triacylglycerol (58:7) levels	IVW	6	1.00(0.99–1.01)	0.641286
116	Phosphatidylcholine (16:0_22:5) levels	IVW	4	1.00(1.00–1.00)	0.649506
117	Phosphatidylcholine (18:0_20:4) levels	IVW	6	1.00(1.00–1.00)	0.659276
118	Phosphatidylinositol (18:0_20:3) levels	IVW	5	1.00(1.00–1.00)	0.65964
119	Phosphatidylcholine (18:1_20:2) levels	IVW	3	1.00(1.00–1.00)	0.660852
120	Phosphatidylcholine (18:0_18:3) levels	WR	1	1.00(0.99–1.01)	0.661876
121	Phosphatidylcholine (O-16:0_18:1) levels	IVW	3	1.00(0.99–1.02)	0.665832
122	Phosphatidylcholine (16:1_20:4) levels	WR	1	1.00(1.00–1.00)	0.668672
123	Phosphatidylethanolamine (O-18:1_20:4) levels	IVW	2	1.00(1.00–1.00)	0.670723
124	Phosphatidylcholine (O-18:0_20:4) levels	IVW	2	1.00(1.00–1.00)	0.697669
125	Phosphatidylcholine (O-16:1_18:1) levels	IVW	3	1.00(0.98–1.01)	0.699428
126	Sterol ester (27:1/17:1) levels	WR	1	1.00(0.98–1.02)	0.703055
127	Sphingomyelin (d38:1) levels	IVW	10	1.00(0.99–1.00)	0.733297
128	Phosphatidylcholine (O-18:1_16:0) levels	IVW	3	1.00(0.99–1.02)	0.735684
129	Phosphatidylcholine (18:0_18:2) levels	IVW	5	1.00(0.99–1.01)	0.742939
130	Sterol ester (27:1/20:3) levels	IVW	6	1.00(1.00–1.00)	0.747485
131	Phosphatidylcholine (18:0_22:6) levels	IVW	2	1.00(1.00–1.01)	0.751353
132	Phosphatidylcholine (18:1_18:2) levels	IVW	5	1.00(1.00–1.01)	0.752776
133	Phosphatidylcholine (16:0_20:5) levels	WR	1	1.00(1.00–1.00)	0.75332
134	Phosphatidylethanolamine (O-16:1_20:4) levels	IVW	2	1.00(1.00–1.00)	0.754466
135	Sphingomyelin (d36:2) levels	IVW	4	1.00(0.99–1.01)	0.761617
136	Phosphatidylethanolamine (O-18:1_18:2) levels	WR	1	1.00(0.99–1.00)	0.781696
137	Phosphatidylcholine (O-18:2_18:1) levels	WR	1	1.00(0.99–1.01)	0.783235
138	Phosphatidylcholine (18:0_20:2) levels	WR	1	1.00(1.00–1.00)	0.787546
139	Phosphatidylcholine (16:0_18:3) levels	IVW	3	1.00(0.99–1.01)	0.789306
140	Sterol ester (27:1/20:5) levels	IVW	3	1.00(1.00–1.00)	0.789929
141	Phosphatidylcholine (O-18:1_18:2) levels	WR	1	1.00(1.00–1.00)	0.791231
142	Phosphatidylethanolamine (18:0_0:0) levels	IVW	2	1.00(0.98–1.02)	0.793894
143	Phosphatidylcholine (16:0_20:4) levels	IVW	4	1.00(1.00–1.00)	0.806426
144	Phosphatidylcholine (14:0_18:2) levels	IVW	2	1.00(0.99–1.01)	0.81781
145	Phosphatidylcholine (15:0_18:2) levels	IVW	5	1.00(0.99–1.01)	0.83309
146	Phosphatidylcholine (16:1_18:1) levels	IVW	5	1.00(0.99–1.01)	0.849638
147	Phosphatidylcholine (O-16:1_20:3) levels	IVW	3	1.00(1.00–1.00)	0.852204
148	Triacylglycerol (56:6) levels	IVW	7	1.00(0.99–1.01)	0.858535
149	Phosphatidylcholine (16:0_0:0) levels	IVW	2	1.00(0.99–1.02)	0.859467
150	Phosphatidylcholine (O-16:0_20:4) levels	IVW	3	1.00(1.00–1.00)	0.867781
151	Phosphatidylinositol (16:0_18:2) levels	IVW	3	1.00(1.00–1.00)	0.870533
152	Phosphatidylcholine (20:4_0:0) levels	IVW	4	1.00(1.00–1.00)	0.879941
153	Phosphatidylcholine (18:1_18:1) levels	IVW	4	1.00(0.99–1.01)	0.901164
154	Phosphatidylinositol (18:0_18:1) levels	IVW	6	1.00(1.00–1.00)	0.907304
155	Phosphatidylinositol (18:1_18:2) levels	IVW	3	1.00(1.00–1.00)	0.910417
156	Phosphatidylcholine (18:1_20:4) levels	IVW	6	1.00(1.00–1.00)	0.950572
157	Phosphatidylcholine (16:1_18:2) levels	IVW	7	1.00(0.99–1.01)	0.961206
158	Phosphatidylcholine (O-18:1_20:4) levels	IVW	4	1.00(0.99–1.01)	0.972677
159	Phosphatidylethanolamine (18:2_0:0) levels	IVW	2	1.00(0.99–1.01)	0.974348
160	Phosphatidylcholine (O-16:1_18:2) levels	IVW	2	1.00(0.98–1.02)	0.979592
161	Phosphatidylcholine (16:0_22:4) levels	IVW	2	1.00(1.00–1.00)	0.987981
162	Sphingomyelin (d40:2) levels	IVW	7	1.00(0.99–1.01)	0.994432

### Identifying key potential lipid metabolites in atherosclerosis

After the crossover of the ADJUSTED < 0.05 fraction of differential lipid metabolites in the above two datasets, we identified six crossover levels of lipid metabolites that affect the risk of atherosclerosis. Finn database:

Cholesterol levels (WR, OR = 2. 310; *P* = 2.063*10–5; [95% CI: 1.970–2.700]), Sterol ester (27:1/18:2) levels (IVW, OR = 1. 410; *P* = 8.977*10–5; [95% CI: 1.210–1.630]), Triacylglycerol (52:4) levels (IVW, OR = 1. 270; P = 9.434*10–5; [95% CI: 1.140–1.410]), Triacylglycerol (52:5) levels (IVW, OR = 1. 340; P = 4.603*10–5; [95% CI: 1.160–1.540]), Diacylglycerol (18:1_18:2) levels (IVW, OR = 1. 220; P = 5.797*10–5; [95% CI: 1.110–1.350]), Triacylglycerol (53:4) levels (IVW, OR = 1.320; P = 7.476*10–5; [95% CI: 1.150–1.510]).

**Fig. 5:**
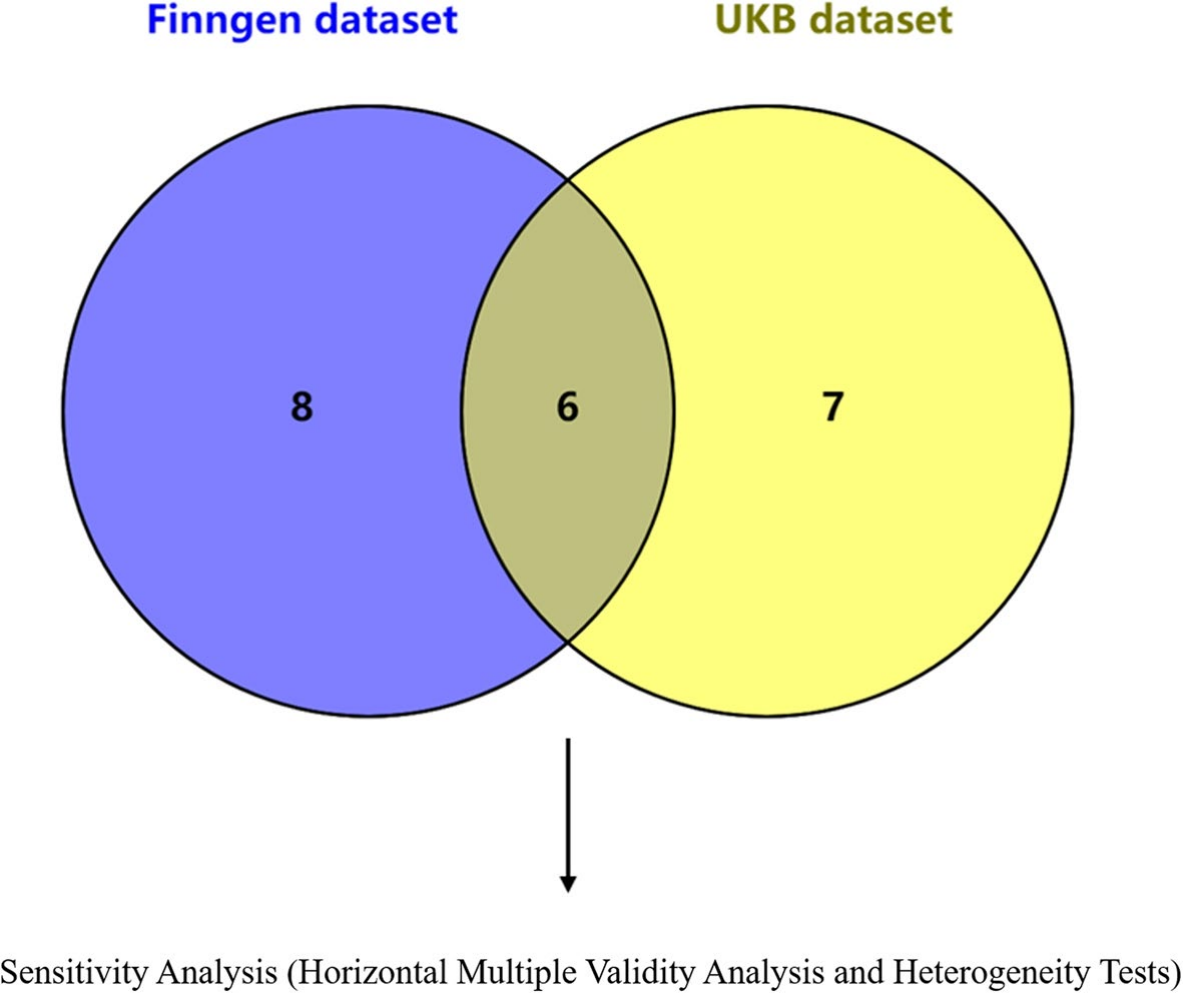
Finngen dataset and UKB dataset take intersection. Sensitivity Analysis (Horizontal Multiple Validity Analysis and Heterogeneity Tests)

UKb database: Cholesterol levels (WR, OR = 1.030; P = 8.444*10–5; [95% CI: 1.020–1.040]), Sterol ester (27:1/18:2) levels (IVW, OR = 1.010; *P* = 7.102*10–5; [95% CI: 1.010–1.020]), Triacylglycerol (52:4) levels (IVW, OR = 1.010; *P* = 0.006*10–5; [95% CI: 1.00–1.010]), Triacylglycerol (52:5) levels (IVW, OR = 1.010; *P* = 2.088*10–5; [95% CI: 1.00–1.010]), Diacylglycerol (18:1_18:2) levels (IVW, OR = 1. 010; P = 1.678*10–5; [95% CI: 1.010–1.010]), Triacylglycerol (53:4) levels (IVW, OR = 1.010; P = 1.776* 10–5; [95% CI: 1.000–1.010]). Detailed information is in Fig. 5, Table 3.

**Table 3:** Intersection between Finngen dataset and UKB dataset of lipid metabolites on atherosclerosis risk

No	id.outcome	Exposure	Method	nSNP	OR(95%CI)	P value	pleiotropy	heterogeneity
1	Finngen dataset	Cholesterol levels	WR	1	2.31(1.97–2.70)	2.06E-25	SNP = 1	SNP = 1
2	Finngen dataset	Sterol ester (27:1/18:2) levels	IVW	9	1.41(1.21–1.63)	8.98E-06	0.4705203	3.40E-19
3	Finngen dataset	Triacylglycerol (52:4) levels	IVW	7	1.27(1.14–1.41)	9.43E-06	0.2601441	0.103666213
4	Finngen dataset	Triacylglycerol (52:5) levels	IVW	4	1.34(1.16–1.54)	4.60E-05	0.960756	5.71E-05
5	Finngen dataset	Diacylglycerol (18:1_18:2) levels	IVW	6	1.22(1.11–1.35)	5.80E-05	0.068707	0.082441293
6	Finngen dataset	Triacylglycerol (53:4) levels	IVW	4	1.32(1.15–1.51)	7.48E-05	0.8654085	0.000136399
7	UKB dataset	Sterol ester (27:1/18:2) levels	IVW	9	1.01(1.01–1.02)	7.10E-05	0.8723856	9.77E-08
8	UKB dataset	Cholesterol levels	WR	1	1.03(1.02–1.04)	8.44E-11	SNP = 1	SNP = 1
9	UKB dataset	Diacylglycerol (18:1_18:2) levels	IVW	6	1.01(1.01–1.01)	1.68E-09	0.4028827	0.105060827
10	UKB dataset	Triacylglycerol (52:4) levels	IVW	7	1.01(1.00–1.01)	3.46E-06	0.8374066	0.115975769
11	UKB dataset	Triacylglycerol (52:5) levels	IVW	4	1.01(1.00–1.01)	2.09E-05	0.3127775	0.382396594
12	UKB dataset	Triacylglycerol (53:4) levels	IVW	4	1.01(1.00–1.01)	1.78E-05	0.4936402	0.294208307

### Verification of MR Presumptions

In our study, SNPs were selected based on the genome-wide significance threshold (*p* < 0.001) in these analyses and no directional pleiotropy was noticed, suggesting that the second MR assumption was not violated (p > 0.05, Table 3). Additionally, the MR heterogeneity test showed no heterogeneity in most of our positive outcomes (*p* > 0.05, Table 3). In summary, the rigorous assessment of the three fundamental assumptions in MR analysis suggested that the selected SNPs were appropriate as genetic instruments, and the relationships between genetically predicted lipid metabolites and atherosclerosis were not influenced by potential confounders or mediators.

### Backtesting the MR hypothesis

Eventually, we concluded that the six lipid metabolites directly affected coronary atherosclerosis, and conversely, we wondered whether the six lipid metabolites increased with the development of coronary atherosclerosis. Therefore, we conducted an MR study with coronary atherosclerosis as an exposure factor and the six lipid metabolites as outcome indicators. The results were all P > 0.05, demonstrating that lipid metabolites did not increase with the development of atherosclerosis.

### MR Analysis: the role of atherosclerosis on lipid metabolites (FinnGen dataset)

Through IVW analyses and weighted median methods, we explored potential causal associations between atherosclerosis and lipid metabolite levels and obtained the following results:

Cholesterol levels (IVW, OR = 0.960; P = 0.472; [95% CI: 0.870–1.060]),

Sterol ester (27:1/18:2) levels (IVW, OR = 1.010; *P* = 0.847; [95% CI: 0.910–1.120]), Triacylglycerol (52:4) levels (IVW, OR = 1.010; *P* = 0.793; [95% CI: 0.930–1.110]),

Triacylglycerol (52:5) levels (IVW, OR = 0.654; *P* = 0.654; [95% CI: 0.940–1.110]),

Diacylglycerol (18:1_18:2) levels (IVW, OR = 1.000; *P* = 0.969; [95% CI: 0.910–1.090]), Triacylglycerol (53:4) levels (IVW, OR = 1.040; *P* = 0.411; [95% CI: 0.950–1.140]).

Detailed information is in Table 4.

**Table 4:** Intersection between Finngen dataset and UKB dataset of atherosclerosis risk on lipid metabolites

No	id.exposure	outcome	exposure	method	P value	estimate
1	Finngen dataset	Cholesterol levels	Coronary atherosclerosis	IVW	0.47174743	0.96(0.87–1.06)
2	Finngen dataset	Sterol ester (27:1/18:2) levels	Coronary atherosclerosis	IVW	0.846917154	1.01(0.91–1.12)
3	Finngen dataset	Diacylglycerol (18:1_18:2) levels	Coronary atherosclerosis	IVW	0.968714183	1.00(0.91–1.09)
4	Finngen dataset	Triacylglycerol (52:4) levels	Coronary atherosclerosis	IVW	0.792861055	1.01(0.93–1.11)
5	Finngen dataset	Triacylglycerol (52:5) levels	Coronary atherosclerosis	IVW	0.653575934	1.02(0.94–1.11)
6	Finngen dataset	Triacylglycerol (53:4) levels	Coronary atherosclerosis	IVW	0.41098834	1.04(0.95–1.14)
7	UKB dataset	Cholesterol levels	Coronary atherosclerosis	IVW	0.895099957	1.18(0.10- 14.33)
8	UKB dataset	Sterol ester (27:1/18:2) levels	Coronary atherosclerosis	IVW	0.975377256	1.04(0.09- 12.42)
9	UKB dataset	Diacylglycerol (18:1_18:2) levels	Coronary atherosclerosis	IVW	0.244369169	0.28(0.03- 2.38)
10	UKB dataset	Triacylglycerol (52:4) levels	Coronary atherosclerosis	IVW	0.429450942	0.42(0.05- 3.59)
11	UKB dataset	Triacylglycerol (52:5) levels	Coronary atherosclerosis	IVW	0.808064609	0.77(0.09- 6.38)
12	UKB dataset	Triacylglycerol (53:4) levels	Coronary atherosclerosis	IVW	0.633706854	1.71(0.19–15.75)

### MR Analysis: the role of atherosclerosis on lipid metabolites (Ukb dataset)

Through IVW analyses and weighted median methods, we explored the potential causal association between atherosclerosis and lipid metabolite levels and obtained the following results:

Cholesterol levels (IVW, OR = 1.180; 95% CI: 0.100–14.330; *P* = 0.895),

Sterol ester (27:1/18:2) levels (IVW, OR = 1.040; 95% CI: 0.090–12.420; *P* = 0.975), Triacylglycerol (52:4) levels (IVW, OR = 0.420; 95% CI: 0.050–3.590; *P* = 0.429),

Triacylglycerol (52:5) levels (IVW, OR = 0.770; 95% CI: 0.090–6.380; *P* = 0.808),

Diacylglycerol (18:1_18:2) levels (IVW, OR = 0.280; 95% CI: 0.030–2.380; *P* = 0.244), Triacylglycerol (53:4) levels (IVW, OR = 1.710; 95% CI:0.190–15.750; *P* = 0.634). Detailed information is in Table 4.

## Discussions

### Main findings

Atherosclerosis is attributed to the abnormal deposition of fibers and lipids throughout the endothelium, which leads to the loss of arterial elasticity and disrupts the vascular structure, resulting in ischemia [14]. The mechanism is that macrophages containing oxidized LDL particles release inflammatory substances, cytokines, and growth factors, which induce cell proliferation and promote leukocyte activation and endothelial dysfunction. Our main finding unveiled that the levels of six lipid metabolites, cholesterol, sterol ester (27:1/18:2), triacylglycerol (52:4), triacylglycerol (52:5), diacylglycerol (18:1_18:2), and triacylglycerol (53:4) directly affected the risk of coronary atherosclerosis. However, their levels did not increase with coronary atherosclerosis.

According to national and international guidelines, elevated low-density lipoprotein cholesterol (LDL-C) is a well-known risk factor for atherosclerotic cardiovascular disease [15]. Our study demonstrated that cholesterol levels were positively associated with the risk of coronary atherosclerosis. Consistently, a study reported in 2020 mentioned that crystalline cholesterol and cholesterol crystals in atherosclerosis were regular features within its necrotic core [16]. Likewise, a study found that Rhodiola rosea glycosides attenuated atherosclerosis in mice by reducing SREBP2 levels and cholesterol and triglyceride biosynthesis [17]. Therefore, lowering cholesterol and triglyceride levels reduces the risk of coronary atherosclerosis, which is exactly in line with the conclusion of our study.

Recently, triglyceride-glucose (TyG) has been considered an index for assessing IR and an important predictor of coronary artery disease severity [18]. It means there is a link between triglyceride levels and coronary atherosclerosis. Consistently, our finding noted that triglycerides reduced the risk of coronary atherosclerosis. A clinical study conducted in 2021 compared the TyG index of 424 patients with NAFLD and 255 patients with coronary artery disease, and it concluded that the TyG index of patients with NAFLD was positively correlated with the risk of coronary artery disease, which may reflect the severity of coronary atherosclerosis [19]. Furthermore, a recent meta-study showed that the incidence of CVD was significantly reduced by controlling factors associated with TyG index or triggers (e.g., blood) that elevate TyG [20]. In conclusion, many of these studies support the present study's conclusion that triglycerides are positively associated with the risk of coronary atherosclerosis.

A recent study showed that long-term consumption of dietary diacylglycerol (DAG) enriched in 1,3-species reduced postprandial lipids, thereby modulating monocyte/macrophage migration and aortic lipid accumulation, eventually alleviating atherosclerosis [21], indicating the correlation between DAG levels and coronary atherosclerosis. Consistently, our study revealed a direct relationship between diacylglycerol kinase (DGK) and the risk of coronary atherosclerosis. Likewise, Toshiki Sasaki et al. elucidated the functional role of DGKα in cardiac injury after ischemia/reperfusion in mouse hearts in vivo and finally concluded that DGKα exacerbated I/R injury by inhibiting the cardioprotective effects of PKCε, ERK1/2, and p70S6K activation [22]. These results suggest that diacylglycerol protein kinase (DGKα) has a positive cardioprotective effect on the heart.

Furthermore, we found that sterol ester levels directly affected the risk of coronary atherosclerosis. Similarly, Avery Sengupta et al. observed the composition, osmotic fragility, and antioxidant status of erythrocyte membranes in normal and hypercholesterolemic rats after consumption of EPA-DHA-rich and ALA-rich sterol esters, and concluded that in cholesterol-rich blood, rat erythrocytes appeared to be deformed and become more fragile. This is because sterol esters can alleviate hypercholesterolemia and thus the risk of coronary atherosclerosis, thereby partially reversing this deformity and fragility [23]. This confirms the conclusion of the present study that sterol ester levels directly influence the risk of coronary atherosclerosis.

There are several strengths of our two-sample MR study. Firstly, we used robust MR analysis methods and selected SNP with strong association as IVs, similar to the experimental framework of a randomized controlled trial. Second, we chose independent, validated genetic variants as IVs to avoid potential confounders and increase the accuracy of our results. Finally, our study pooled many MR studies and ultimately screened out the most significant six lipid metabolites, providing a theoretical basis for the clinical treatment of atherosclerosis.

### Limitations

However, our study has limitations. The GWAS data include only European people, so further studies are needed to determine the generalizability of our findings to different populations. In addition, there are gender differences in the prevalence of coronary atherosclerosis. Unfortunately, the public databases from which our data were obtained do not allow for detailed subgroup analyses for specific demographics (e.g., age and sex).

### Future research

In the future, we will conduct more experiments to investigate the correlation between lipid metabolites and coronary atherosclerosis, with a focus on lipid metabolites that are directly related to the occurrence and development of coronary atherosclerosis, such as cholesterol and triglycerides. The intricate nature of lipid metabolism and its metabolites is not fully understood, underscoring the necessity for additional basic and clinical studies. Improving lipid metabolites presents a promising avenue for addressing coronary atherosclerosis. These six lipid metabolites have the potential as new biomarkers for predicting the risk of atherosclerosis, providing new insights into the treatment and prevention of cardiovascular diseases.

## Conclusions

In conclusion, our study comprehensively elucidates the causal relationship between lipid metabolites and the risk of coronary atherosclerosis. Cholesterol levels, sterol ester (27:1/18:2) levels, triacylglycerol (52:4) levels, triacylglycerol (52:5) levels, diacylglycerol (18:1_18. 2) levels, and triacylglycerol (53:4) levels are positively correlated with the risk of coronary atherosclerosis onset. However, the levels of these six lipid metabolites do not increase with the development of coronary atherosclerosis.

## Data Availability

All data generated or analyzed during this study are included in this published article (and its Supplementary Information files).

## Abbreviations

CVD: Cardiovascular disease
ACS: Acute coronary syndrome
HF: Heart failure
SCD: Sudden cardiac death
MR: Mendelian randomization
SNPs: Single nucleotide polymorphisms
IVs: Instrumental variables
LD: Linkage disequilibrium
IVW: Inverse variance-weighting
ORs: Odds ratios
Cis: Confidence intervals
LDL-C: Low-density lipoprotein cholesterol
TyG: Triglyceride-glucose
DGK: Diacylglycerol kinase
